# Probing coordinated co-culture cancer related motility through differential micro-compartmentalized elastic substrates

**DOI:** 10.1038/s41598-020-74575-y

**Published:** 2020-10-28

**Authors:** Szu-Yuan Chou, Chang-You Lin, Theresa Cassino, Li Wan, Philip R. LeDuc

**Affiliations:** 1 Departments of Mechanical Engineering, Biomedical Engineering, Computational Biology, and Biological Sciences, Carnegie Mellon University, Pittsburgh, PA 15213 USA; 2 Departments of Physics, Carnegie Mellon University, Pittsburgh, PA 15213 USA

**Keywords:** Lab-on-a-chip, Cancer microenvironment

## Abstract

Cell development and behavior are driven by internal genetic programming, but the external microenvironment is increasingly recognized as a significant factor in cell differentiation, migration, and in the case of cancer, metastatic progression. Yet it remains unclear how the microenvironment influences cell processes, especially when examining cell motility. One factor that affects cell motility is cell mechanics, which is known to be related to substrate stiffness. Examining how cells interact with each other in response to mechanically differential substrates would allow an increased understanding of their coordinated cell motility. In order to probe the effect of substrate stiffness on tumor related cells in greater detail, we created hard–soft–hard (HSH) polydimethylsiloxane (PDMS) substrates with alternating regions of different stiffness (200 and 800 kPa). We then cultured WI-38 fibroblasts and A549 epithelial cells to probe their motile response to the substrates. We found that when the 2 cell types were exposed simultaneously to the same substrate, fibroblasts moved at an increased speed over epithelial cells. Furthermore, the HSH substrate allowed us to physically guide and separate the different cell types based on their relative motile speed. We believe that this method and results will be important in a diversity of areas including mechanical microenvironment, cell motility, and cancer biology.

## Introduction

A cell microenvironment contains information in the form of both chemical and mechanical properties. The chemical microenvironment is biologically relevant through influencing growth, differentiation, and apoptosis in a diversity of cells^1,2^. However, the influence of mechanical properties on cells presented with competing mechanical and differential environments, such as those found in the microenvironments of tumors, remains poorly understood. There is increasing interest in understanding the role of microenvironmental mechanics in order to develop novel therapies to target this factor in cancer pathology^3–5^.

Cell mechanics in general has been expanding at a rapid rate as cells throughout the body have been found to be heavily affected by their micro-mechanical environments^6–8^. Cells in the body develop in a wide range of mechanical contexts already, such as soft neuronal tissue or hard bone tissue^9–11^. On the microenvironmental scale, cells experience isometric and tensional forces generated by cell–cell and cell-extracellular matrix (ECM) interactions^12–14^. The mechanical properties of these substrates alter a range of cell processes, including cell differentiation, as cells tune their responses to the specific tissues in which they reside. For example, soft matrices are conducive to neural cell survival and differentiation^15^, whereas harder matrices can result in cell differentiation towards osteogenic responses^16^. Beyond differentiation, the mechanical context of cells is involved in the pathogenic progression of cancer as well^17–19^. When normal mammary epithelial cells transition to become a malignant breast tumor, those cells become increasingly stiffer^20,21^. Surprisingly, reducing substrate stiffness was sufficient to revert tumors towards a non-malignant phenotype^20,21^.

In many of the cell responses including cancer, motility is one of the main cell responses affecting their overall phenotypic responses^21–23^. Motility is already known to be affected by changes in mechanical substrate stiffness. For example, NIH 3T3 fibroblasts were guided by the rigidity of the substrate during movement^24^. Fibroblasts cultured on flexible polyacrylamide sheets coated with type I collagen, migrated preferentially to the soft region when faced with a mechanically different substrate boundary. Gray et al.^25^ also reported that NIH/3T3 cells and bovine pulmonary arterial endothelial cells accumulated preferentially on PDMS substrates with higher stiffness. Soft lithography was used to micropattern PDMS substrates. Cells detected the mechanical cues of the substrate, which altered their response during migration. Here, we present our approach for probing co-cultured cells as a model of tumor cell response to localized substrate elasticity. To accomplish this, we utilized a previously described process to microfabricate composite polydimethylsiloxane (PDMS) substrates comprised of regions of distinct stiffness that were “harder” or “softer” (in this work: 800 kPa or 200 kPa, respectively) to create a hard soft hard (HSH) surface system^25–27^. In order to model two major cell types in tumors, malignant epithelial cells and fibroblasts, we studied the effects of substrate stiffness on WI-38 fibroblasts and A549 epithelial cells independently and in coordination. We isolated the contribution of these two cell types by first observing individual cell type responses to substrates of varying stiffness. We then compared this behavior to the coordinated response of co-cultured cell types that were allowed to interact with the substrate and with each other. In the co-culture system, the WI-38 fibroblasts moved more toward the soft area of the HSH substrate when compared to A549 epithelial cells, indicating the HSH system may be useful for separation of different cell types. In addition, we compared the motility of cells on the HSH substrate with uniform substrates and observed that both cell types migrated longer in the direction of the soft channel during controlled time periods. Our approach indicates that in addition to cell separation, the HSH substrate may be able tto spatially and mechanically guide cells for controlled motility in co-culture.

## Materials and methods

### Fabrication of substrates with differential stiffness

**Figure 1:**
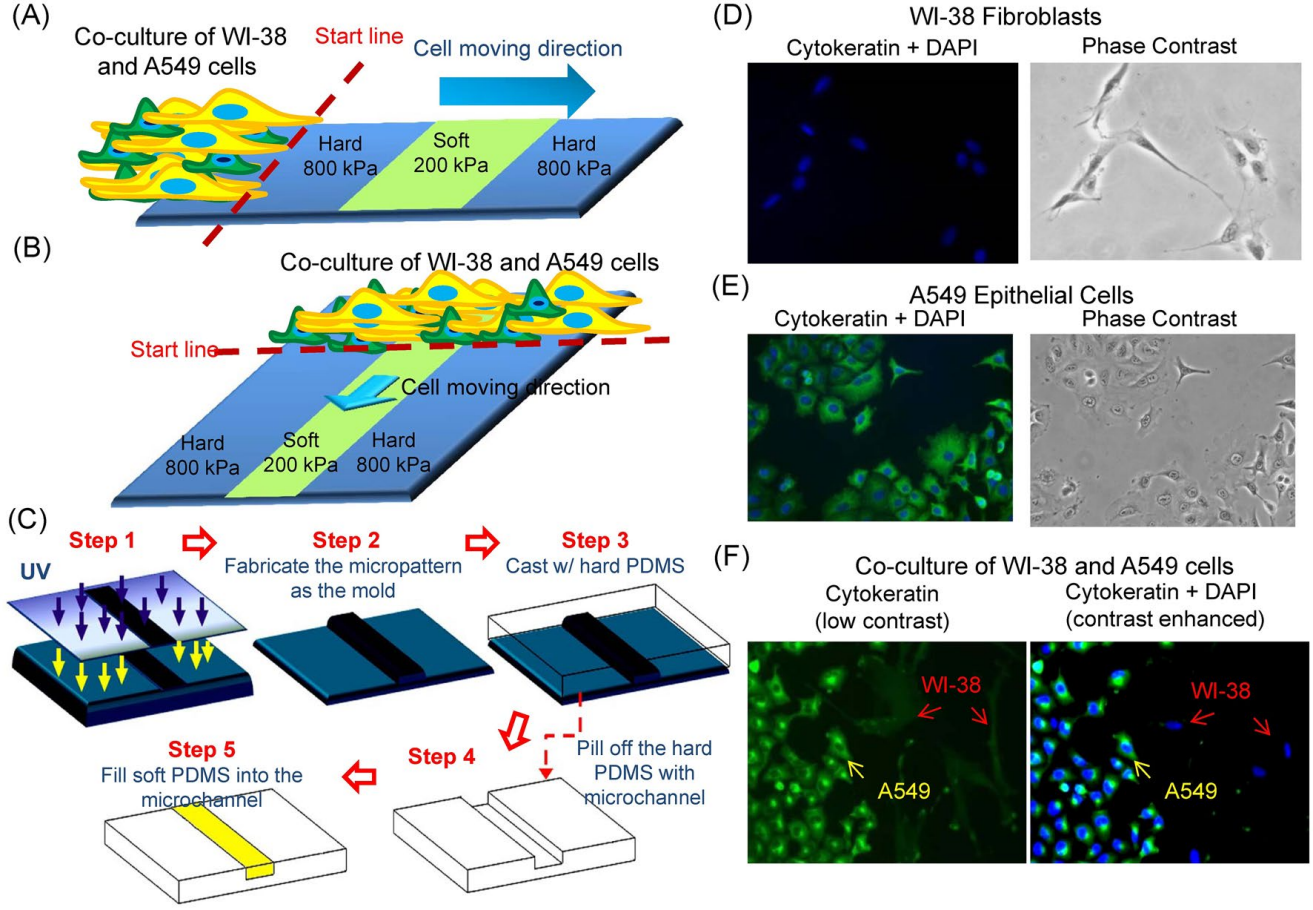
Probing coordinated co-culture tumor cell related motility through differential micro-compartmentalized elastic substrates. (**A**,**B**) Schematics of our hard–soft–hard (HSH) substrate to probe the effects of localized stiffnesses on cell motility for co-cultures. The soft region is depicted in green and the hard region in blue. (**A**) For the horizontal configuration, the cells were released from the side of the system moving across the width of the rectangular stipe of the HSH substrate. (**B**) For the vertical configuration, the cells were released from the top of the HSH substrate and moved along the direction of the stripe. (**C**) A schematic of the fabrication process for the hard–soft–hard substrate^28^. We first fabricated the microstructure substrate through conventional soft lithography by exposing photoresist on a silicon wafer to UV light (step 1). We create a mold with microstructures of desired dimension (step 2) and then cast PDMS with a 5:1 ratio of base to curing agent against the mold (step 3) to create the hard substrate. After the PDMS cured, we peeled the microchannel from the mold (step 4) and then poured PDMS with a 30:1 ratio of base/curing agent for the soft polymer into the microchannels (step 5). We cured the system at room temperature for at least 48 h producing the final substrate with micro-patterned differential elasticities. (**D**–**F**) A549 epithelial cells and WI-38 fibroblasts immunofluorescent stained with Cytokeratin (green) and DAPI (blue). (**D**) Cytokeratin and DAPI staining with solely (**D**) WI-38 fibroblasts, (**E**) solely A549 epithelial cells; both Cytokeratin and DAPI are positive. (**F**) Cytokeratin and DAPI staining for localized co-culture of WI-38 fibroblasts and A549 epithelial cells.

To probe the effect of local variance in stiffness on coordinated cell motility, a polymer composite system of patterned differential elasticities was created^28^ for cell culture with different cell types (Fig. 1A,B). Specifically, polymeric microchannels were fabricated using conventional soft lithography, and then filled with PDMS of different stiffness (Fig. 1C). Substrates with varying stiffness were prepared by mixing hard base with soft curing agents in different ratios: 1:50 (10 kPa), 1:30 (200 kPa), and 1:5 (800 kPa). The result was a surface with alternating stiffness of 800 kPa and 200 kPa where the “soft surface” (with a surface stiffness of 200 kPa) was a long rectangular strip 50–100 μm in width in the middle of the “hard surface” (with a surface stiffness of 800 kPa) forming a hard–soft–hard system (HSH). Similarly, the hard surface could be used for the long rectangular strip surrounded by the soft surface creating a soft–hard–soft system (SHS).

### Preparation of substrate for culture

PDMS substrates were sterilized using 70% ethyl alcohol (diluted with deionized water from 190 proof, 95%, ACS/USP grade; PHARMCO-AAPER, Inc, Brookfield, CT, USA; No: 111USP190) and then washed with filtered Phosphate-buffered saline (PBS; Fisher Scientific International, Inc, Hampton, NH, USA; No: BP399-500). PDMS substrates were coated with extracellular matrix for 60 min to enhance cell attachment. The extracellular matrix was either fibronectin (10 μg/mL PBS; BD Biosciences, San Jose, CA, USA; No.: 39410) or collagen type I (Sigma, diluted to 0.01% with deionized water). After coating, the extracellular matrix solution was washed out and the substrates were dried in the cell culture hood for 20 min.

### Cell motility experimental preparation and control

**Figure 2:**
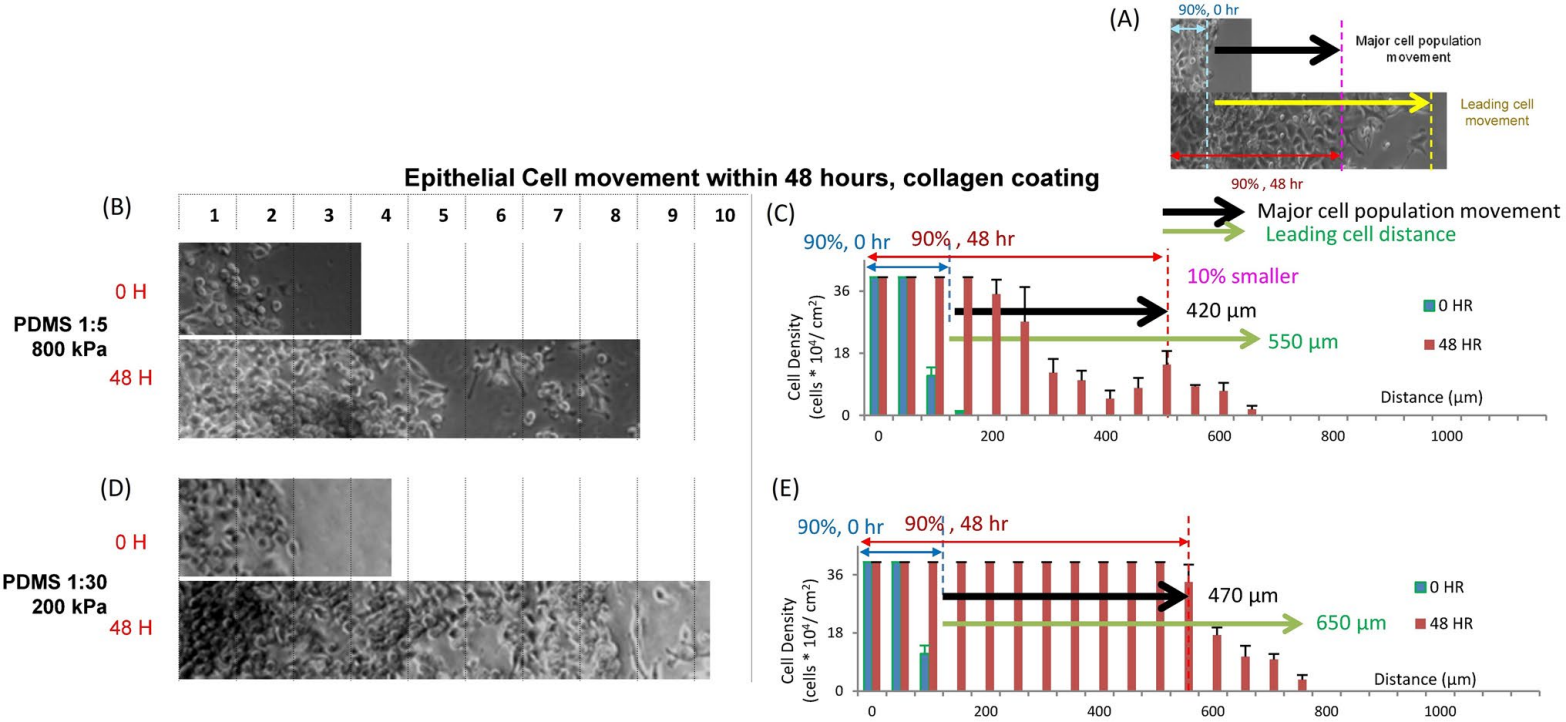
The motility of A549 epithelial cells on collagen coated PDMS substrates with different stiffnesses. (**A**) A diagram to indicate collagen coated PDMS substrates with different stiffnesses. Images and quantitation, respectively, of the movement of A549 epithelial cells attached on PDMS substrates with stiffnesses including (**B**,**C**) 800 kPa and (**D**,**E**) 200 kPa at 0 h and 48 h after they were released. Data are standard deviation with total cell counts = 4379 and n = 3.

**Figure 3:**
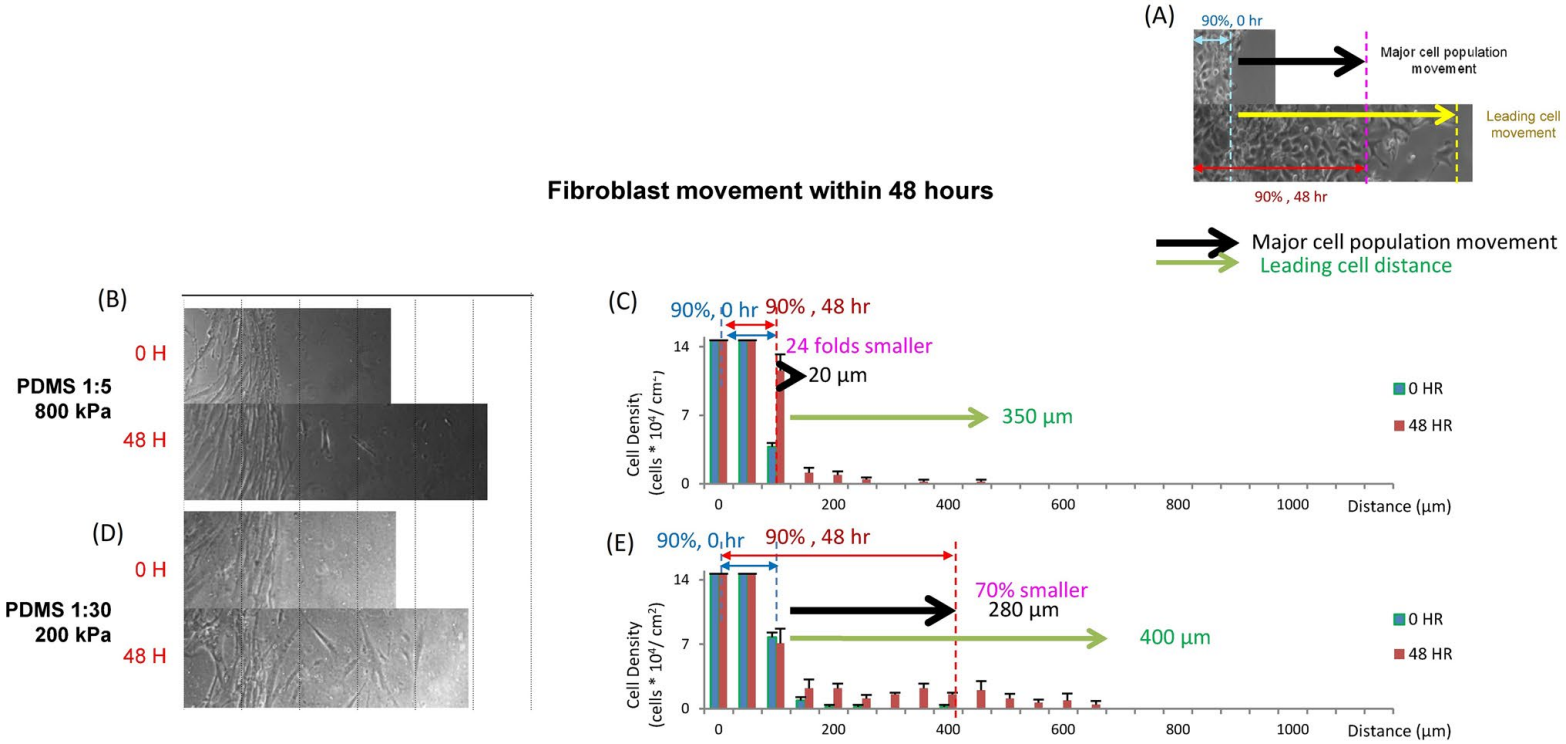
The motility of WI-38 fibroblast cells on fibronectin coated PDMS substrates with different stiffnesses. (**A**) A diagram to indicate “major cell population movement” and “leading cell distance”. Images and quantitation, respectively, of the movement of A549 epithelial cells attached on fibronectin coated PDMS substrates with stiffnesses including (**B**,**C**) 800 kPa and (**D**,**E**) 200 kPa at 0 h and 48 h after they were released. Data are standard deviation with total cell counts = 1470 and n = 3.

Human WI-38 fibroblast and A549 human lung adenocarcinoma epithelial cells (American Type Culture Collection) were cultured on our polymer composite substrates with different stiffness values. The cells were cultured at 37 °C in media supplemented with 10% fetal bovine serum (ATCC) and 1% Penicillin–Streptomycin (Mediatech). Cells were incubated for a minimum of 6 h to allow for them to attach and spread. In order to obtain a consistent controlled starting region (i.e. starting line) for the cell motility response and analysis, the cells were first confined by covering half of the substrate surface by a slab of PDMS. This PDMS slab was composed of base to curing agent ratios 1:30. After applying the PDMS membrane to block the cell culture regional attachment and spreading during cell culture, the cells including WI-38 fibroblasts and A549 epithelial cells were seeded for the motility experiments with either single cell type or co-culture experiments. After the cells reached approximately 100% confluence, the cell response in motility on substrates with singular elasticity was initiated, and the constraining PDMS slabs were removed. This created a cell free zone on the surface of the substrates, with a clear starting line of the locations of the cells (Fig. 1). After removing the PDMS slab, the culture media was refreshed to remove suspended cells. The cell movement was observed by taking images at 0-h, 24-h, or 48-h on the surface of the substrates in order to track the leading edge of the cell population movement. The distances of the cell movement were measured for different cell-substrate combinations (Figs. 2, 3, Supplementary Figures S1–S3).

### Fluorescent staining and data analysis

In order to identify the A549 epithelial cells from WI-38 fibroblasts, the co-culture samples were immunofluorescently labeled with DAPI and Cytokeratin solutions (containing 1:1:1 of diluted MS X Cytokeratin AE1/AE3 antibody (Chemicon International MAB3412) 1/160 in Antibody Diluent (ScyTek, #ABB500), Anti-Cytokeratin antibody [1/2.7 in Antibody Diluent, Becton Dickinson, Cat # 349205) and Anti-Cytokeratin 7 mouse monoclonal antibody (1/2.7 in Antibody Diluent, BioGenex REF AM255-5 M)]. Cells were first washed with PBS, and then fixed. Cells were then washed with PBS and then incubated with the Cytokeratin solution for 1 h at the room temperature. Next, the cells were washed with PBS and then incubated with a second antibody Alexa Fluor 488 goat anti-mouse IgG (Molecular Probes) for 1 h at the room temperature. The samples were finally washed, mounted with DAPI Fluoromount-G (SouthernBiotech), and imaged using an inverted fluorescent microscope (Zeiss, Axiovert 200). The experimental results of the fibroblasts and epithelial cells on different substrates were analyzed by taking cell images at time 0-h, 24-h (for co-culture), or 48-h (for single cell types). The WI-38 fibroblasts were negative to Cytokeratin staining while the A549 epithelial cells were positive to Cytokeratin (Fig. 1E). In addition, the morphology of WI-38 fibroblasts was distinct from the A549 epithelial cells. The leading cell movement represented the longest distance that one cell traveled relative to the starting line. In addition, a 90% distribution approach was implemented, which analyzed the location of 90% of the cells relative to the initial starting line. This approach allowed us to understand the migration of leading cells with respect to the majority cell movement, preventing outliers from skewing the leading cell movement analysis. Student T tests and Chi-square tests were performed to compare the motility results.

## Results and discussion

To study coordinated cell motility with localized elasticity substrates, we first fabricated a substrate with localized alternating elasticities to control the mechanical microenvironment of the cells. We implemented two experimental methods for our cell motility experiments with HSH substrates: horizontal HSH experiments, where cells moved across the width of the strip regions, and vertical HSH experiments, where cells moved along the length of the strip regions (Fig. 1A,B). For the horizontal HSH approach (Fig. 1A), the cells were released by removing the constraining PDMS slab from the side allowing cells to migrate across the width of the strip; the cells did not physically interact with the soft strip before releasing. For the vertical HSH setup (Fig. 1B), the cells were cultured at the top of the HSH substrate. The cells were already attached to both the hard and the soft region of the HSH substrate before releasing in the vertical HSH set up, but the PDMS constraining slab kept them all spatially constrained until released.

We first investigated the motility of A549 epithelial cells and WI-38 fibroblasts attached on uniform substrates with different stiffnesses over time. The stiffnesses were 10 kPa, 200 kPa and 800 kPa^29,30^. We analyzed the motility of A549 epithelial cells (Fig. 2, Supplementary Figure S1) with respect to the farthest cell movement. We were careful to analyze leading cells using the 90% cell distribution method, which described the movement of leading cells pertaining to the “majority” movement, preventing outlier cells from skewing the data. The epithelial cells had the greatest 90% cell motility when released on collagen coated PDMS substrates with 200 kPa rigidity after 48 h (Fig. 2D,E). For the epithelial cells attached to the 200 kPa substrate, the 90% cell distribution was 470 μm (Fig. 2E). Cells attached to the 10 kPa substrates moved 40% less than those attached on 200 kPa substrates (Supplementary Figure S1E). The 90% cell distribution for cells attached on the 800 kPa substrates was 10% less than those attached on 200 kPa substrates (Fig. 2C), and for cells attached on the glass was 70% smaller than those attached on 200 kPa substrates (Supplementary Figure S1C). The farthest cell movement of the epithelial cells was approximately the same distance (650 μm) for cells attached on different substrates. The exception was epithelial cell movement on the 800 kPa substrates (Fig. 2C), which was 20% smaller. Our results show that within 48 h we were able to detect discernable differences in the epithelial cell response to uniform substrate rigidity at three distinct stiffnesses ranging from 10 to 800 kPa.

We then examined the motility of epithelial cells and fibroblasts with substrates coated in fibronectin (Supplementary Figure S3, Fig. 3). Epithelial cells moved the least distance on 800 kPa fibronectin coated substrates which was consistent with our findings from collagen coated substrates. However, unlike collagen coated substrates, the 10 kPa fibronectin coated substrates (Supplementary Figure S3H,I) showed the largest cell movement when compared to the other groups. The movements of epithelial cells attached to the 200 kPa fibronectin coated substrates (Supplementary Figure S3F,G) were 20% smaller when compared to the cells attached on 10 kPa substrate (Supplementary Figure S3I). This suggests that different ECM coatings could a major factor affecting the motility of epithelial cells. For fibroblasts, the 800 kPa substrates induced the least motility for distance after 48 h (Fig. 3B,C). The 90% distribution group was almost zero with only a few cells moving as little as 350 μm. Fibroblasts attached to softer substrates showed much greater motility (10 kPa, Supplementary Figure S2D,E) with 90% of the cells moving 500 μm within 48 h. This speed was similar to the epithelial cell movement on 10 kPa fibronectin coated substrates (Supplementary Figure S3H,I). Furthermore, fibroblasts attached to harder substrates, such as glass and high-stiffness (800 kPa) PDMS, tended to move collectively (Figs. 3B, 3C, Supplementary Figure S2B,C), while those attached to lower-stiffness (200 kPa and 10 kPa) PDMS substrates tended to move more discretely, with single cells moving further than the group more often (Figs. 3D, 3E, Supplementary Figure S2D,E). These behaviors were quantified as different distribution tendencies in cell density. Fibroblasts attached glass (Supplementary Figure S2C) and 800 kPa PDMS (Fig. 3C), showed a large decrease (around 80%) of cell density at specific distances (100–150 μm for glass, and 150–200 μm for 800 kPa PDMS) while fibroblasts attached 200 kPa PDMS (Fig. 3E) and 10 kPa PDMS (Supplementary Figure S2E), showed cell densities that were relatively smooth.

**Figure 4:**
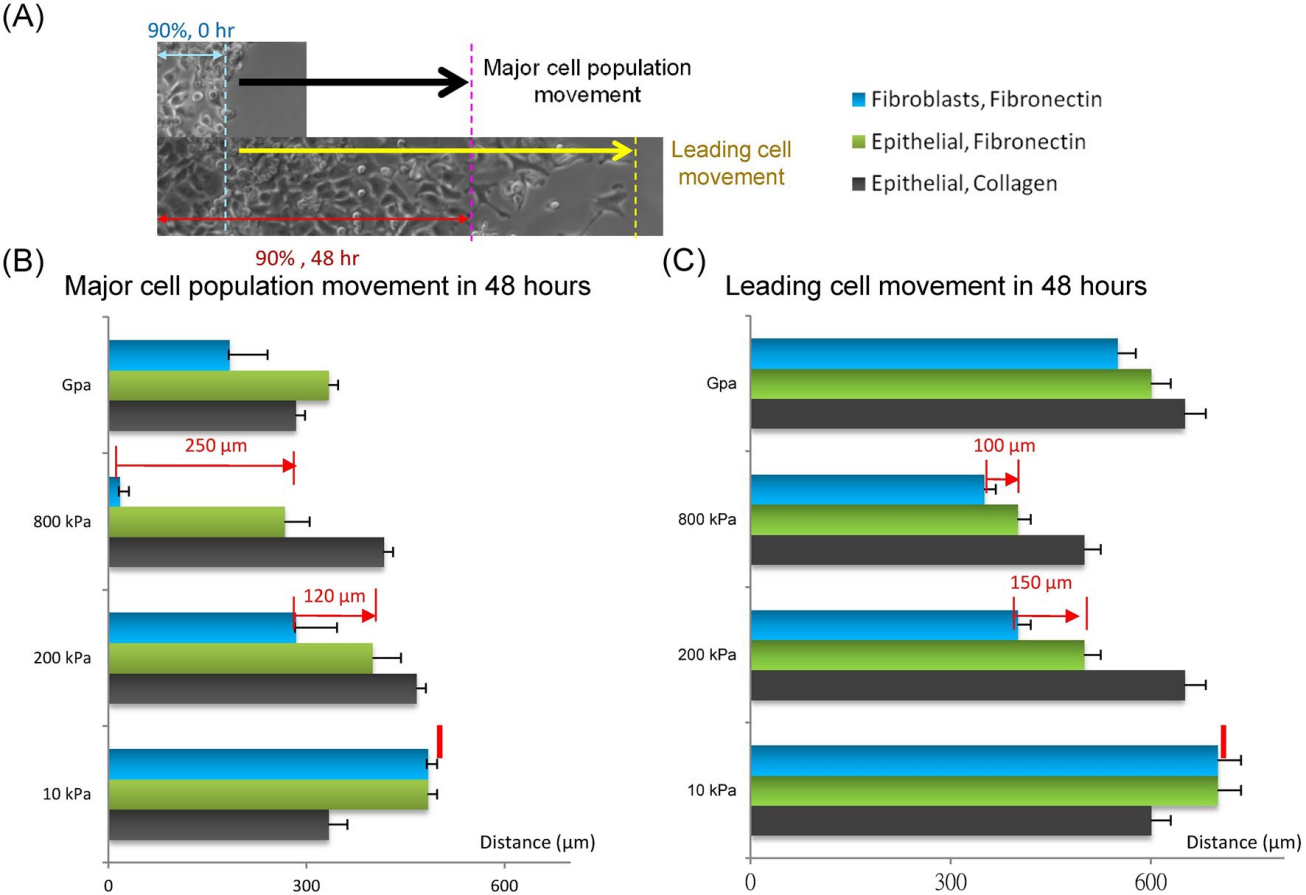
Comparison of cell motility results for A549 epithelial cells and WI-38 fibroblasts on different substrates: (**A**) A diagram to indicate “Major cell population movement” and “leading cell distance”. (**B**) A comparison of cell motility results for the major cell population movement for A549 epithelial cells attached on fibronectin coated PDMS substrates, A549 epithelial cells attached on collagen coated PDMS substrates, and WI-38 fibroblasts attached on fibronectin coated PDMS substrates with stiffnesses including 10 kPa, 200 kPa, 800 kPa and glass. Data are standard deviation. (**C**) A comparison of cell motility results with the leading cell movement for A549 epithelial cells attached on fibronectin coated PDMS substrates, A549 epithelial cells attached on collagen coated PDMS substrates, and WI-38 fibroblasts attached on fibronectin coated PDMS substrates with stiffnesses from 10 kPa, 200 kPa, 800 kPa and glass. The difference in cell response between the two cell types to substrate stiffness is statistically significant (P values < 10^–5^ between the epithelial cells and the fibroblasts for both the 90% distribution and the leading cell movement).

We then compared the cell motility on varying substrate stiffnesses for epithelial cells and fibroblasts that were cultured separately (Fig. 4). The fibroblasts on the 800 kPa and the 200 kPa substrates moved 250 μm and 120 μm, respectively for the 90% cell movement (Fig. 4B), and 100 μm and 150 μm, respectively in leading cell movement (Fig. 4C). We then selected the 200 kPa substrate as our “Soft substrate”, and the 800 kPa substrate as our “Hard substrate" for the following experiments.

Cell motility has been found to have mixed responses to substrate stiffnesses. In the high stiffness range (> 20–50 kPa in general), further increases in stiffness can inhibit cell migration. Shukla et al.^31^ reported decreases of A549 cell migration speeds with an increase in PDMS stiffness from 27 to 4756 kPa, which is similar to our results. In addition, decreased cell motility relative to increasing stiffness has been reported in other findings^32–35^. This response may be due to an effect in which high rigidity substrates prevent cells from mechanistically remodeling their actin and focal adhesions robustly^36^.

**Figure 5:**
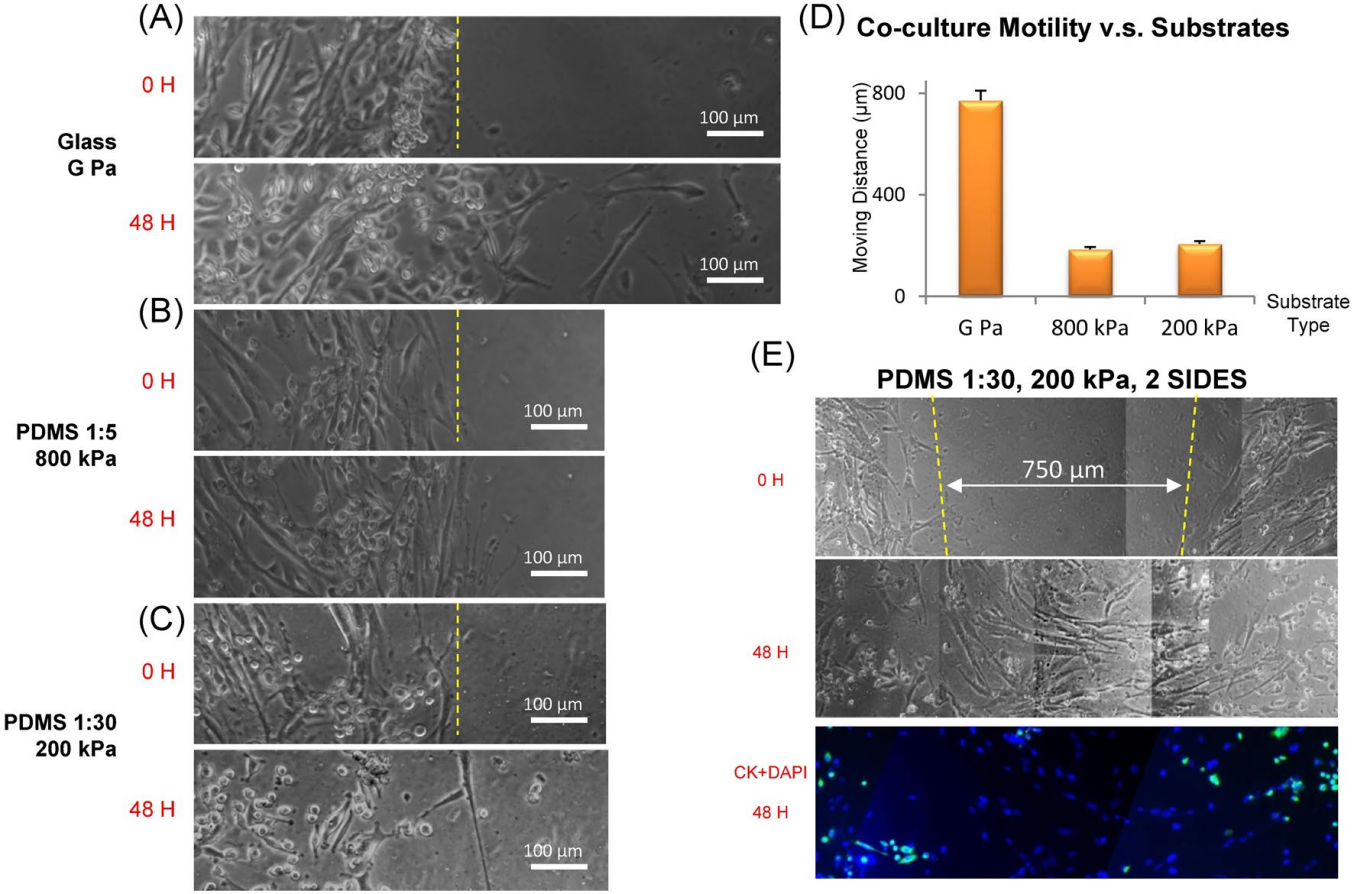
The response of A549 epithelial cells and WI-38 fibroblasts co-cultured together with respect to PDMS substrates with different rigidities after 24 h. (**A**–**C**) The phase contrast images for cells attached on the substrates after being released for 0-h and at 48-h for (**A**) glass, (**B**) 800 kPa and (**C**) 200 kPa. (**D**) The leading-edge cell movement of each cell-substrate combination. Data are standard deviation. (**E**) The response of A549 epithelial cells and WI-38 fibroblasts co-cultured together after a PDMS slab was released created an open section lacking cell between the two culture areas (left and right) after 0 and 48 h. Immunofluorescence composite image captured 24 h after the cells were released. The fibroblasts moved into the open region within 24 h while the epithelial cells did not. Green pseudo-coloring for was for Cytokeratin and A549 epithelial cells and blue pseudo-coloring was for DAPI and the nucleus. When comparing the cell movement attached on glass to 200 kPa or 800 kPa PDMS, the P values were less than 5 × 10^–3^. However, the P value was 0.2 for comparing the cells on the 200 kPa and the 800 kPa PDMS substrates, indicating no statistically significant difference.

We then investigated cell motility for a co-culture system containing epithelial cells and fibroblasts attached to uniform substrates. Figure 5A–C shows the co-cultured response to uniform PDMS substrates with a stiffness of 200 kPa and 800 kPa compared to glass after 24 h. We found that the movement of co-culture cells on both the 200 kPa and 800 kPa PDMS substrates was less than 30% in comparison to glass (Fig. 5D). The cells attached to the 800 kPa PDMS substrates moved collectively compared to the cells attached to the 200 kPa substrates in which cells tended to travel further from the group individually. We also observed cells cultured on the 200 kPa substrates with a wound-like, cell-free space between the two cell populations (the space between the yellow dot lines in Fig. 5E). The cells filled the wound-like space up to 750 μm wide within 24 h with this space being most occupied by the fibroblasts (the blue dots in the fluorescent image of Fig. 5E). Collective cell migration is regulated by a complex series of cell signaling and migrations that generally occurs in morphogenesis, tissue repair and cancer^37^. When cells were distributed on the substrate with a narrow open space in between, they would migrate collectively with higher motility than cells collectively on a substrate^38,39^ (Fig. 5C). Murrell et al.^40^ discovered that the free space with no wound creation or cell death is sufficient to induce a wound-healing response, which is similar to our findings. However, this phenomenon was not significant for cells attached on either the 800 kPa or glass substrates.

**Figure 6:**
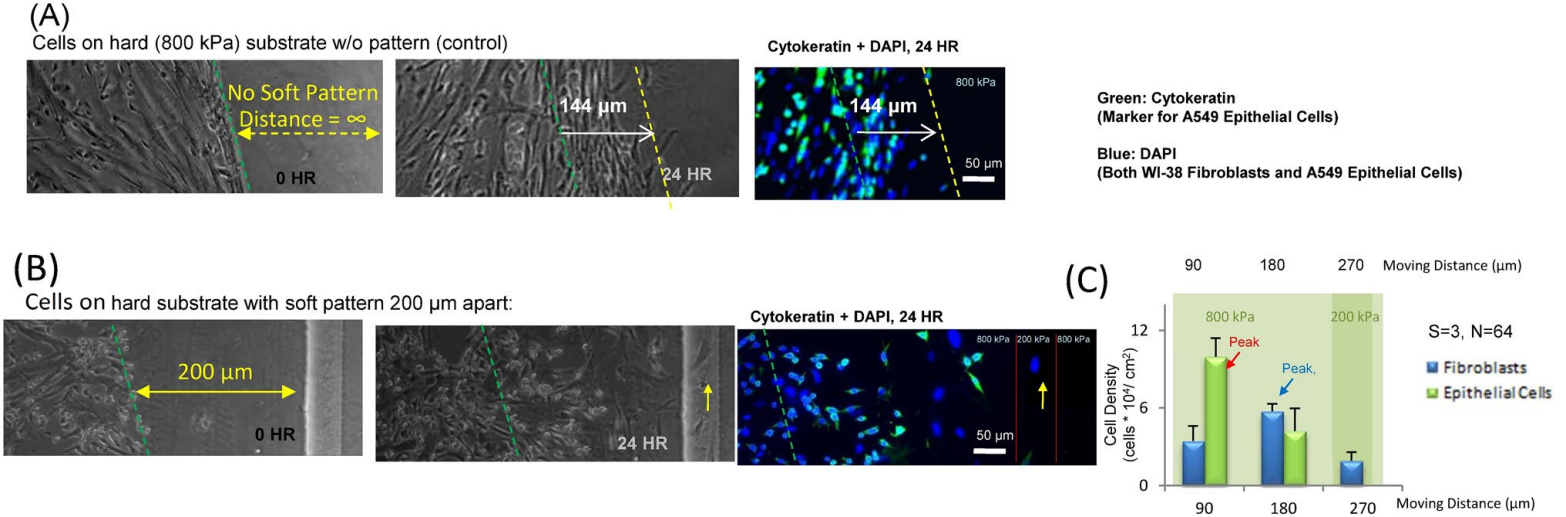
A549 epithelial cells and WI-38 fibroblasts co-cultured motility for the horizontal configuration with cells moving across the width of the rectangular strip of our HSH system. (**A**) The co-culture was released and recorded after 24 h for the 800 kPa PDMS substrate with no soft region as a control. (**B**) The co-culture was released at a distance of 200 μm away from the closest edge of the soft pattern and recorded after 24 h, and then the movement was quantified. Yellow arrow indicates the cell that traveled the longest distance in the image. (**C**) Green pseudo-coloring for was for Cytokeratin and A549 epithelial cells and blue pseudo-coloring was for DAPI and the nucleus with n = 3 and a total cell count of 66. The P value comparing the fibroblasts and the epithelial cells distribution after 24 h was 0.00067 for 200 μm.

Our next step was to create a composite substrate with localized different stiffness to investigate the movement of the fibroblasts and the epithelial cells when co-cultured together. We utilized fibronectin coating on PDMS in the co-culture experiments for comparison to single cell experiments (Fig. 3; Supplementary Figure S3). Based upon the results of our previous experiments with different stiffnesses, we used 200 kPa and 800 kPa as our soft and hard substrate. We first implemented our co-culture HSH horizontal setup as a control (Fig. 6A). Then we released the co-cultured cells 200 um away from the soft pattern (Fig. 6B). We observed that fibroblast and epithelial cells showed different migration features with respect to their population distributions. Epithelial cells were found at a higher density further from the release point (90 μm). At a 180 μm distance just before the hard-soft interface, the density of fibroblasts and endothelial cells were similar. At a location beyond the interface position (270 μm), the fibroblasts were the only cell type found, indicating that after the soft interface, the fibroblasts were still moving across the interface.

**Figure 7:**
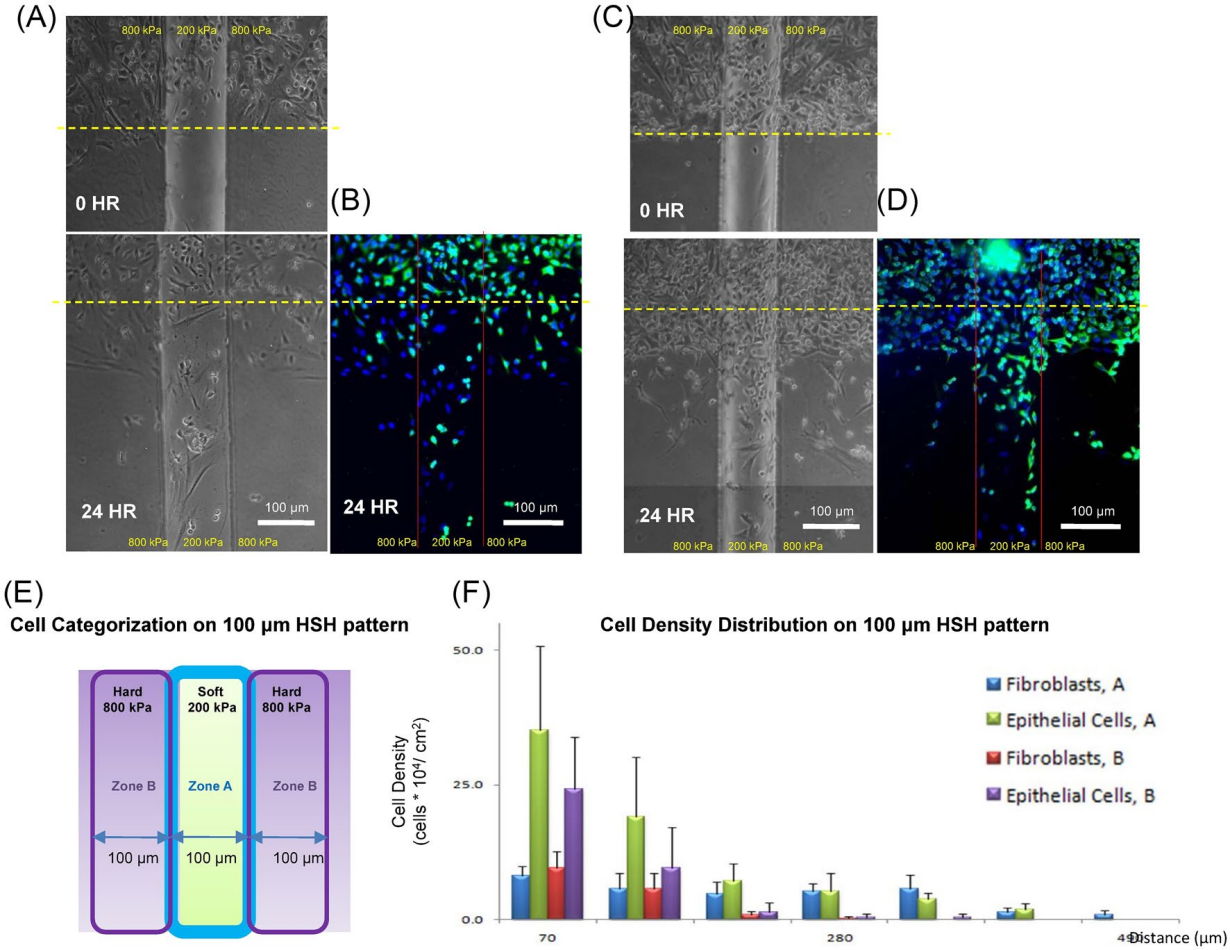
A549 epithelial cells and WI-38 fibroblasts co-cultured motility for the vertical configuration with cells moving along the length of the rectangular strip with a width of 100 μm in our HSH system. (**A**) The co-culture was imaged with phase contrast at 0 h and 48 h after being released. (**B**) Immunofluorescent images were captured after 24 h as well. A second representative response of the A549 epithelial cells and WI-38 fibroblast response at (**C**) 0 and 48 h, and (**D**) with immunofluorescence. (**E**) A diagram indicating the location of zone A and zone B on the HSH substrate for (**F**) quantifying the motility of the A549 epithelial cells and WI-38 fibroblasts on the vertical HSH substrate. Green pseudo-coloring for was for Cytokeratin and A549 epithelial cells and blue pseudo-coloring was for DAPI and the nucleus. The differences between the two cell types were statistically significant (P value = 10^–24^) for the cells located inside the 100 μm wide soft strip (group A).

We next examined the response of the co-cultured cells to the HSH vertical setup (Fig. 1B) with a 100 μm wide, soft rectangular region (Fig. 7). Two representative images show the movement of the co-cultured cell types in response to differential substrate stiffnesses (Fig. 7A–D). Cells that migrated more than 150 μm from the release line (the yellow dot lines in Fig. 7A–D) traveled either inside the soft rectangular region or outside the soft region but still within 100 μm from the edge of the soft region. The co-cultured cell movement appeared to be guided towards and along the localized soft region. For the cells that migrated more than 150 μm from the release point, almost no cells were located more than 100 μm away from the edge of the soft region. We used this observation to categorize the cells into groups A or group B for quantification (Fig. 7E). The cells located inside the soft region were categorized into group A, and the cells located 100 μm away from the soft region were categorized into group B. The results of the HSH substrate with a 100 μm soft region showed fibroblasts to have a more consistent response compared to epithelial cells after 24 h. While there was no prominent physical separation of the two cell types within 24 h, we did observe migration tendencies between the two cell types as they traveled along the HSH substrate to be distinct (Fig. 7F). For the fibroblasts inside the soft region (“Fibroblasts, A” in Figure S3F), the cell density remained almost constant (5.2 ± 10% × 10^4^ cells/cm^2^) from 70 μm to 350 μm away from the starting point. The density of fibroblasts then started to decrease on the soft region but could migrate up to 450 μm after only 24 h compared to the density of epithelial cells which steadily decreased as they traveled on the soft region (Fig. 7F).

**Figure 8:**
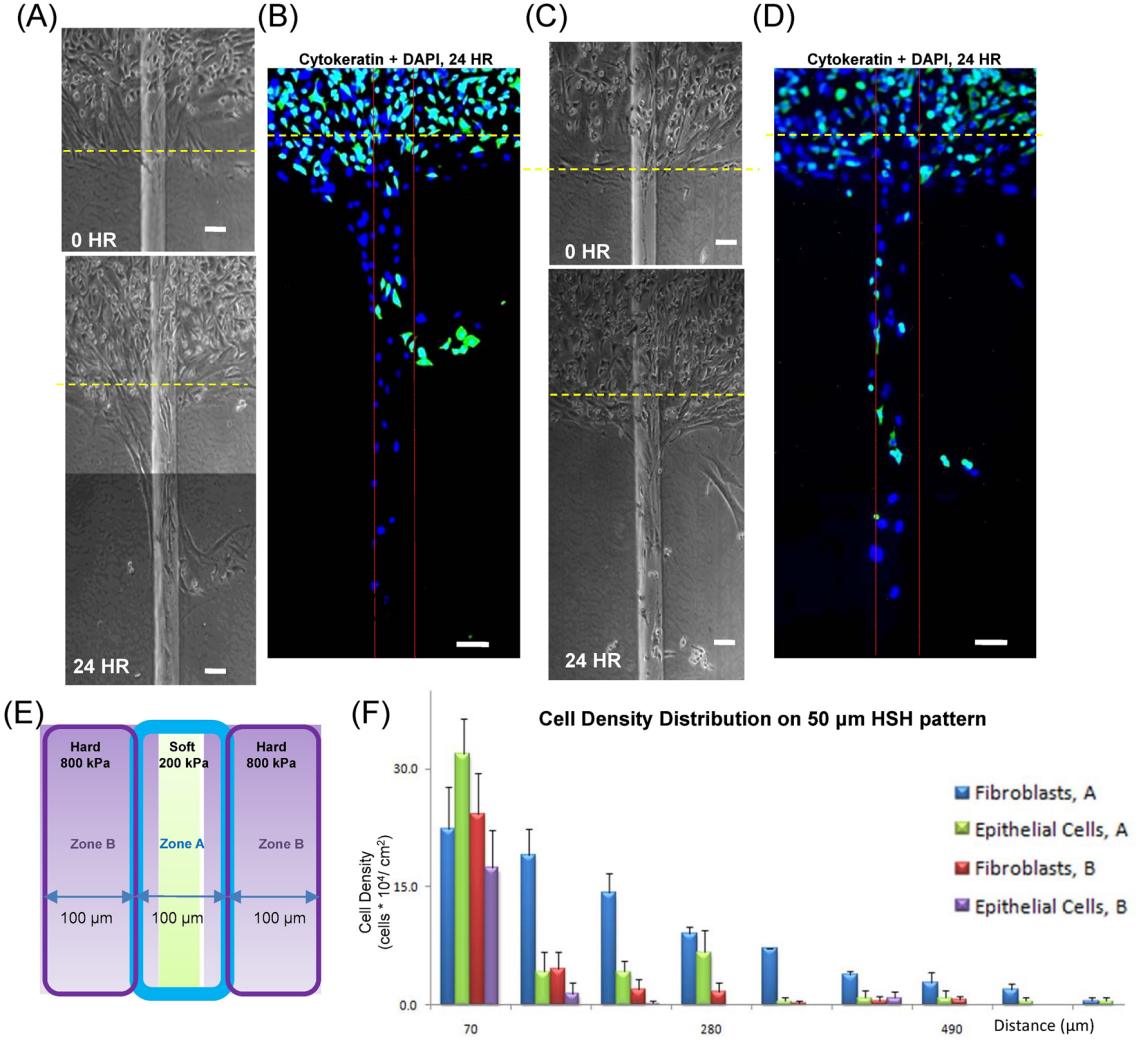
A549 epithelial cells and WI-38 fibroblasts co-cultured motility for the vertical configuration with cells moving along the length of the rectangular strip with a width of 50 μm in our HSH system. (**A**) The co-culture was imaged with phase contrast at 0 h and 48 h after being released. (**B**) Immunofluorescent images were captured after 24 h as well. A second representative response of the A549 epithelial cells and WI-38 fibroblast response at (**C**) 0 and 48 h, and (**D**) with immunofluorescence. (**E**) A diagram indicating the location of zone A and zone B on the HSH substrate for (**F**) quantifying the motility of the A549 epithelial cells and WI-38 fibroblasts on the vertical HSH substrate. Green pseudo-coloring for was for Cytokeratin and A549 epithelial cells and blue pseudo-coloring was for DAPI and the nucleus. The fibroblasts and epithelial cells on the HSH substrate were statistically significant (P value is 0.01 for zone A and 0.2 for zone B) and thus less than 0.05.

Based on results in Fig. 7, we hypothesized that the motility difference could be utilized for cell separation by manually changing the geometry of the soft region of the HSH substrate. Thus, we next conducted motility experiments of the co-cultured cells on the vertical HSH substrate with a 50 μm wide soft strip (Fig. 8). After 24 h, the cell population was observed to be more localized for the 50 μm soft strip (Fig. 8). Cells that migrated more than 100 μm from the release line did not travel farther than 100 μm away from the soft strip. This result reflects the ability of our HSH geometry to guide cell movement for the co-culture of A549 epithelial cells and WI-38 fibroblasts along the localized soft strip. By categorizing the cells into group A or group B as previously discussed (Fig. 8E), we determined that the cells on the soft strip (group A) traveled up to 600 μm. The cells that were close to the soft strip (group B) traveled up to 450 μm. For cells with no considerable interaction with the soft strip (i.e. cells that were not in group A and group B) we observed movement of less than 100 μm within the 24-h time frame. Overall, the co-cultured cells migrated a large distance (600 μm) within a short time frame (24 h) compared to our previous cell motility experiments described in this study. Furthermore, we observed that the co-cultured cells showed a higher difference in motility based on cell type when attached to the HSH substrate with the 50 μm soft strip, indicating the potential of the geometry to serve as a cell type separator. On the soft strip, over 80% of the cell population located at the 70–140 μm region from the starting line (the yellow dot lines in Fig. 8A–D) (group A), were fibroblasts. Conversely, the density of epithelial cells in group A decreased by over 85% at the same location when compared to the epithelial cells located at the 0–70 μm region from the starting line. Our results showed a significant rate of separation. A full separation cannot be expected in our experiments as epithelial cells are likely to collectively interact with fibroblasts in the cell culture in a similar fashion to their natural interactions physiologically. In addition, when comparing the 50 μm strip versus the HSH 100 μm soft region approaches, cells on the 50 μm soft strip moved 600 μm, which is 30% greater than the cell migration distance on the 100 μm soft region HSH substrate (450 μm). The cells have better overall motility with the 50 μm soft strip on the HSH substrate compared to the 100 μm soft region. From the results in the vertical HSH motility experiments, the differences of cell movement between the fibroblasts and epithelial cells at zone A were statistically significant, at zone B however, there were no significant differences between the two cell types.

**Figure 9:**
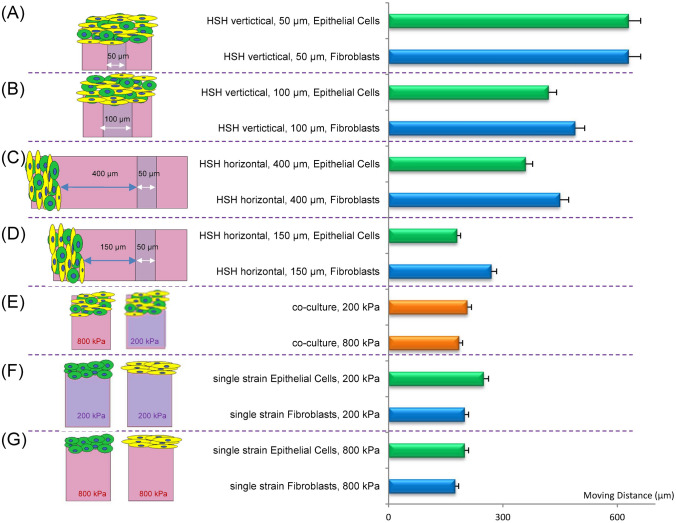
A summary of the cell motility results. The leading cell movement from 0 to 24 h for (**A**) co-cultured cells on the HSH vertical with a 50 μm wide stripe, (**B**) co-cultured cells on the HSH vertical set up with a 100 μm wide stripe, (**C**) co-cultured cells on the HSH horizontal at a distance of 400 μm from the stripe, (**D**) co-cultured cells on the HSH horizontal at a distance of 150 μm from the stripe, (**E**) co-cultured cells on a uniform substrate with stiffnesses of either 800 kPa or 200 kPa, (**F**) just A549 epithelial cells or WI-38 fibroblasts on uniform substrate with a stiffness of 200 kPa, and (**G**) just A549 epithelial cells or WI-38 fibroblasts on uniform substrate with a stiffness of 800 kPa. The single cell type results were divided by 2 from the 48 h leading cell experiments, to approximate a comparison to the 24-h time points.

Figure 9 summarizes the motility results for either the single strain cell culture or the co-culture cells response to the different substrates including the uniform stiffness substrates and the HSH substrates. Overall, we observed that the cells attached to the HSH substrates had higher distances of movement after 24 h when compared to the cells attached to uniform substrates. Furthermore, although the motility responses were different for fibroblasts and epithelial cells on the HSH substrates, both cell types had greater movement on the HSH substrate. These results indicate the difference in motility due to cell types and cell substrates, but also partially due to the different distributions of the two cell types between substrates of various stiffness. Crosstalk between fibroblast and epithelial cells has been recognized as a common response in tumor progression^41,42^. Many studies have discovered cancer-associated fibroblasts enhance the motility of cancer cells through multiple regulatory signals including α-smooth muscle actin, SMAD family number-3 (SMAD3), cyclin-dependent kinase inhibitor-1, miR-29b, etc.^41,43,44^. While our study was mainly conducted on PDMS substrates with higher stiffness than found in vivo, we discovered an increase in epithelial cell motility in co-culture only on a HSH substrate, i.e. when a difference of stiffness existed. Further research in the fibroblast-epithelial interactions and regulation signals would be interesting to provide more information on environmental effects relative to these mechanisms.

## Conclusion

We developed an approach to examine the motility of co-cultured cells with differential substrate stiffnesses. We implemented our approach by exposing WI-38 fibroblasts and A549 epithelial cells to PDMS substrates with different stiffnesses from 10 to 800 kPa. This approach could be a future model for tumor biopsies, which are often composed of mixed cell types including epithelial cells and fibroblasts. Single cell types have distinct responses to PDMS substrates with different stiffnesses, and we observed the slowest movement to be on the 800 kPa PDMS substrate. When fibroblasts and epithelial cells were co-cultured to examine their response to different uniform substrate stiffnesses, their responses became less statistically significant. We then created a polymer composite system to impose localized elasticity control and used it to examine the motility of co-cultured fibroblasts and epithelial cells. Through our approach, the co-cultures exhibited distinct behavior according to their location on the polymer composite system and their cell type. Both fibroblasts and the epithelial cells were observed to have increased motility when they were on our polymer composite system compared to the cells attached to the uniform substrate. The fibroblasts were observed to have a higher cell density on the soft substrate than the epithelial cells through our approach. This approach could be useful in a variety of areas including cell-substrate interactions, mechanobiology, cell motility, and cell separation.

## Supplementary information

Supplementary Information.
