# Supplementary material for: Probing coordinated co-culture cancer related motility through differential micro-compartmentalized elastic substrates
Source: Sci Rep. 2020 Oct 28;10:18519. doi: 10.1038/s41598-020-74575-y (PMC7595178; doi:10.1038/s41598-020-74575-y)
Supplement: Supplementary file 1 — Supplementary Information. [file 41598_2020_74575_MOESM1_ESM.docx]

# probing coordinated co-culture cancer related motility through differential micro-compartmentalized elastic substrates

Szu-Yuan Chou^1^, Chang-You Lin^2^, Theresa Cassino^1^, Li Wan^1^, and Philip R. LeDuc^1^

^1^ Departments of Mechanical Engineering, Biomedical Engineering, Computational Biology, and Biological Sciences, Carnegie Mellon University, Pittsburgh, PA 15213, USA

^2^ Departments of Physics, Carnegie Mellon University, Pittsburgh, PA 15213, USA

***Keywords***: Motility; Localized elasticity; Cell-substrate interactions; Soft lithography; Metastasis


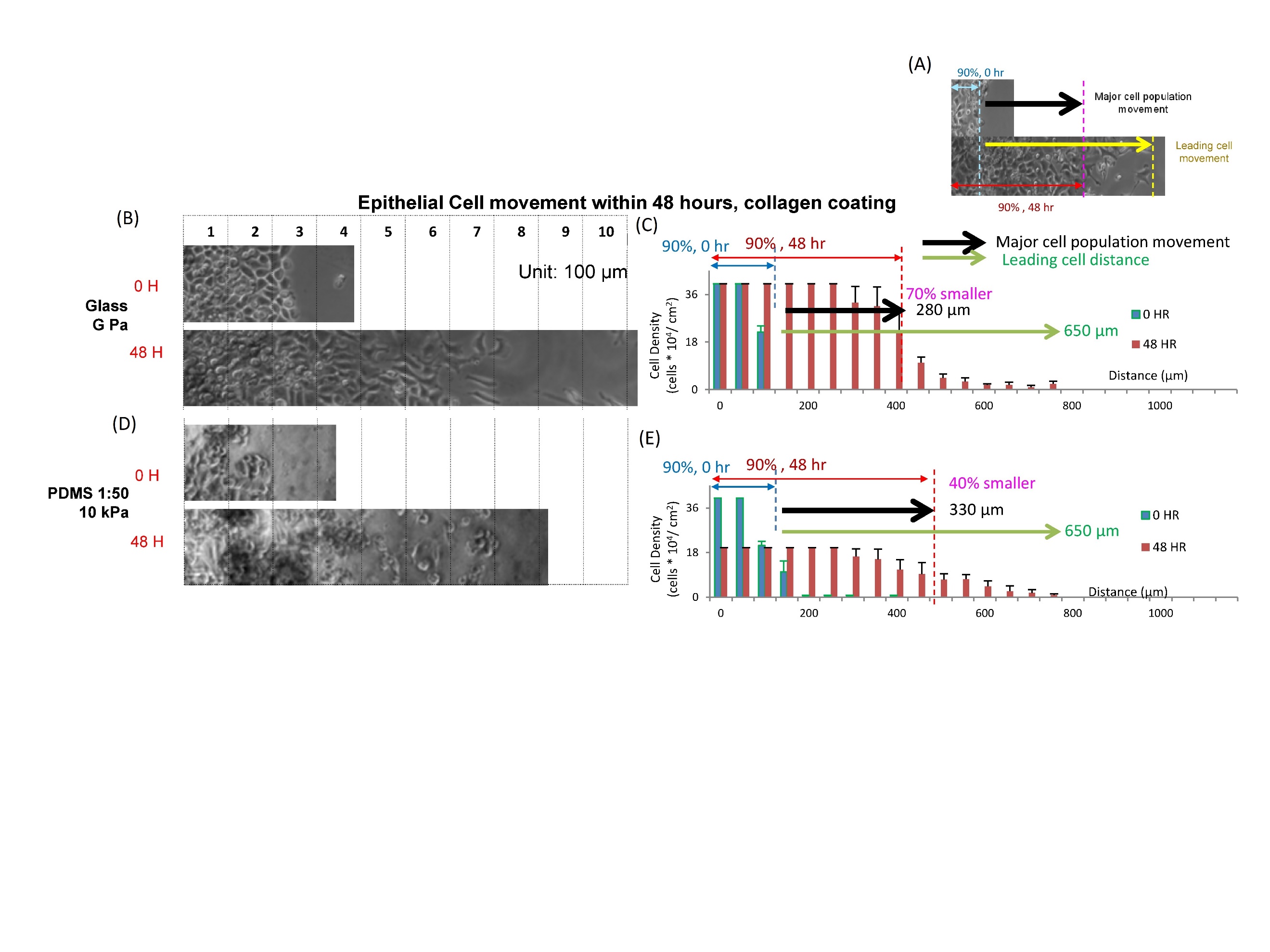
 Figure S1. The motility of A549 epithelial cells on collagen coated PDMS substrates with different stiffnesses. (A) A diagram to indicate collagen coated PDMS substrates with different stiffnesses. Images and quantitation, respectively, of the movement of A549 epithelial cells attached on PDMS substrates with stiffnesses including (B, C) glass and (D, E) 10 kPa at 0 hours and 48 hours after they were released. Data are standard deviation with total cell counts =4379 and n=3.


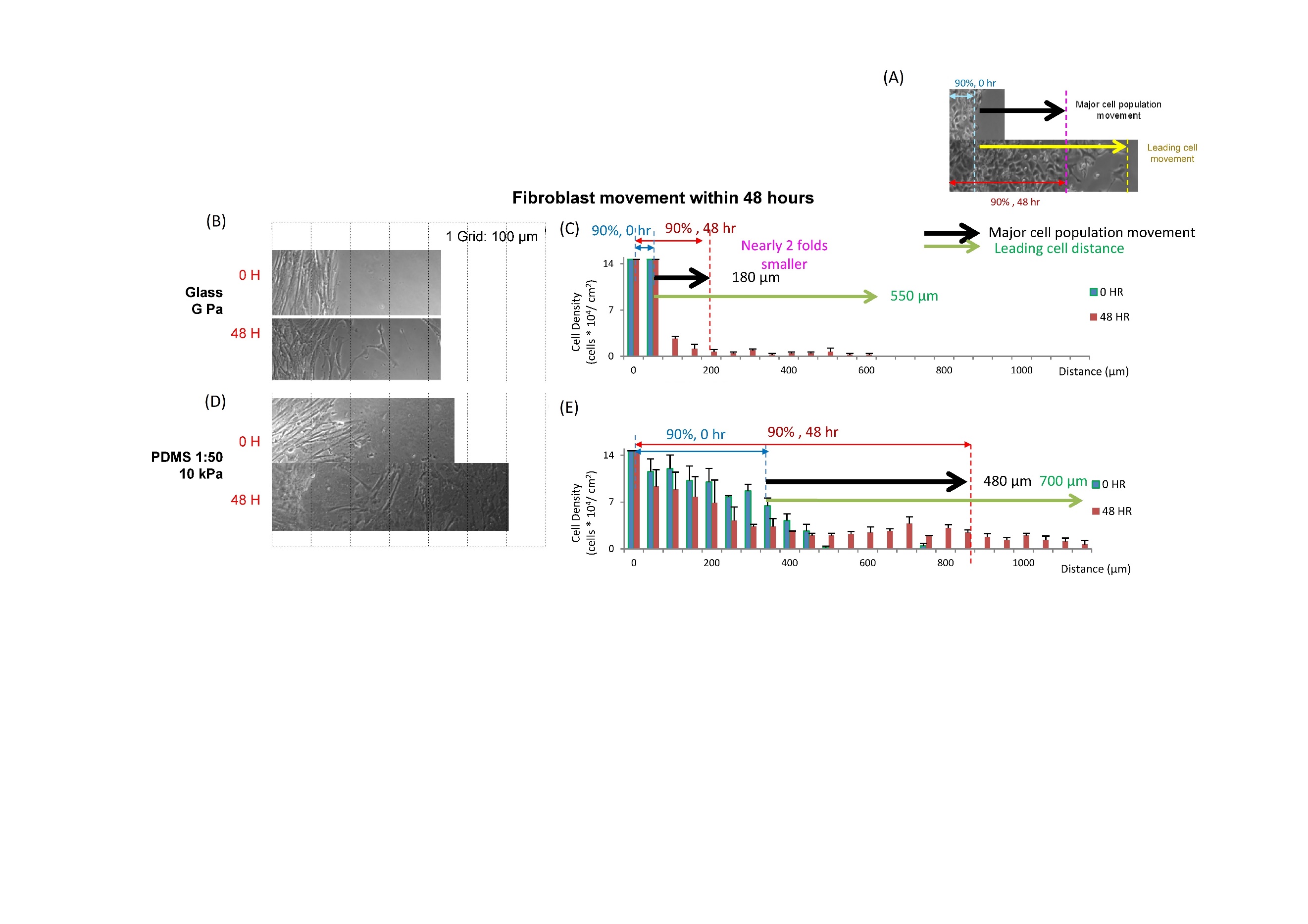
 Figure S2. The motility of WI-38 fibroblast cells on fibronectin coated PDMS substrates with different stiffnesses. (A) A diagram to indicate “major cell population movement” and “leading cell distance”. Images and quantitation, respectively, of the movement of A549 epithelial cells attached on fibronectin coated PDMS substrates with stiffnesses including (B, C) glass and (D, E) 10 kPa at 0 hours and 48 hours after they were released. Data are standard deviation with total cell counts =1470 and n=3.


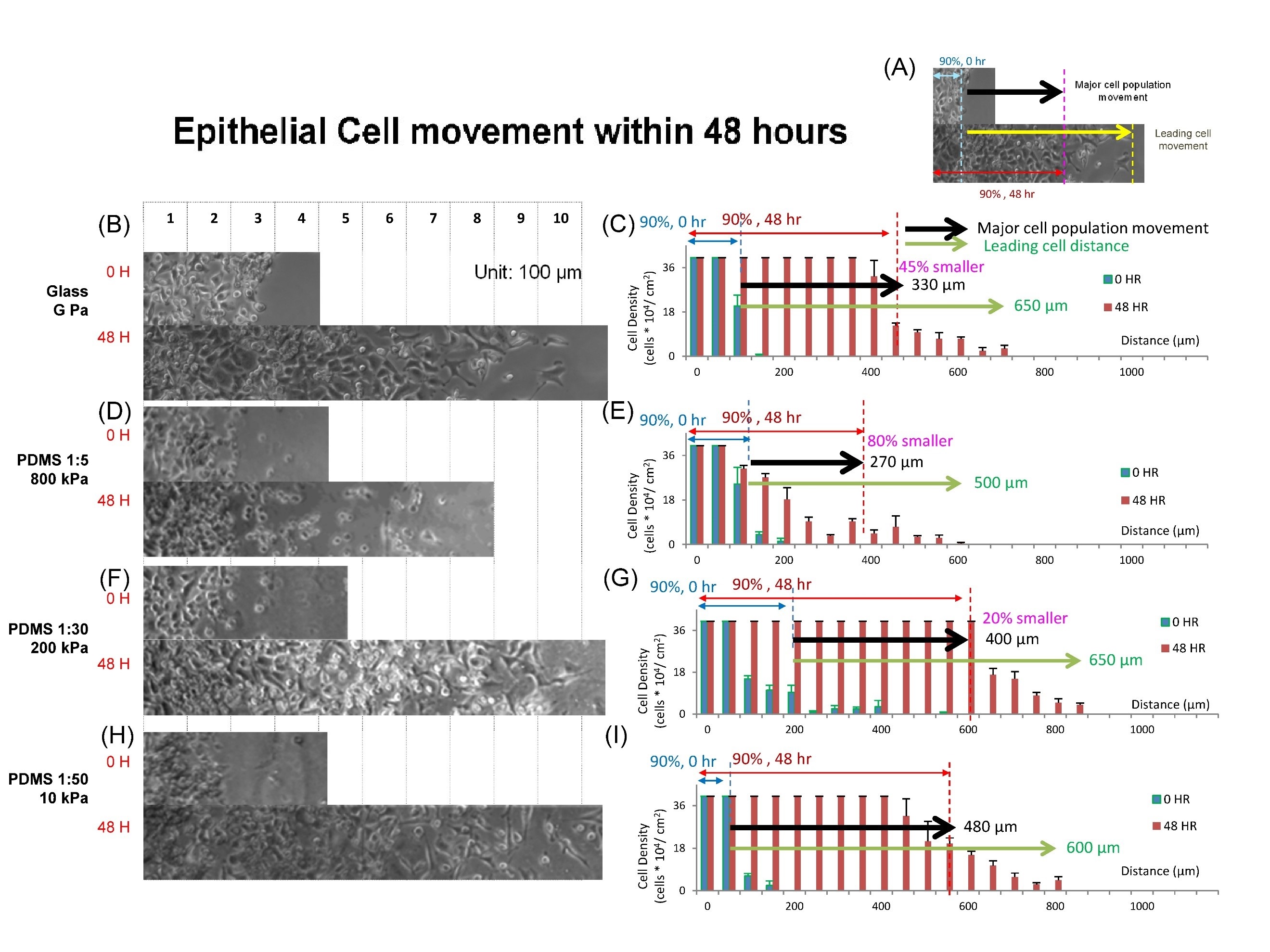


Figure S3. The motility of A549 epithelial cells on fibronectin coated PDMS substrates with different stiffnesses. (A) A diagram to indicate PDMS substrates with different stiffnesses. (A) A diagram, images and quantitation, respectively, of the movement of A549 epithelial cells attached on fibronectin coated PDMS substrates with stiffnesses including (B, C) glass, (D, E) 800 kPa, (F, G) 200 kPa and (H, I) 10 kPa at 0 hours and 48 hours after they were released. Data are standard deviation with total cell counts =4372 and n=3.
